# Identification of Gα_12_-vs-Gα_13_-coupling determinants and development of a Gα_12/13_-coupled designer GPCR

**DOI:** 10.1038/s41598-024-61506-4

**Published:** 2024-05-15

**Authors:** Manae Tatsumi, Christian Cruz, Nozomi Kamakura, Riku Kuwabara, Gaku Nakamura, Tatsuya Ikuta, Ravinder Abrol, Asuka Inoue

**Affiliations:** 1 Graduate School of Pharmaceutical Sciences, Tohoku University, 6-3, Aoba, Aramaki, Aoba-ku, Sendai, Miyagi 980-8578 Japan; 2 Department of Chemistry and Biochemistry, California State University, Northridge, CA 91330 USA; 3 Present Address: Graduate School of Pharmaceutical Sciences, Kyoto University, 46-29 Yoshida-Shimo-Adachi-cho, Sakyo-ku, Kyoto, 606-8501 Japan

**Keywords:** Pharmacology, Molecular modelling, Drug discovery

## Abstract

G-protein-coupled receptors (GPCRs) transduce diverse signals into the cell by coupling to one or several Gα subtypes. Of the 16 Gα subtypes in human cells, Gα_12_ and Gα_13_ belong to the G_12_ subfamily and are reported to be functionally different. Notably, certain GPCRs display selective coupling to either Gα_12_ or Gα_13_, highlighting their significance in various cellular contexts. However, the structural basis underlying this selectivity remains unclear. Here, using a Gα_12_-coupled designer receptor exclusively activated by designer drugs (DREADD; G_12_D) as a model system, we identified residues in the α5 helix and the receptor that collaboratively determine Gα_12_-vs-Gα_13_ selectivity. Residue-swapping experiments showed that G_12_D distinguishes differences between Gα_12_ and Gα_13_ in the positions G.H5.09 and G.H5.23 in the α5 helix. Molecular dynamics simulations observed that I378^G.H5.23^ in Gα_12_ interacts with N103^2.39^, S169^3.53^ and Y176^34.53^ in G_12_D, while H364^G.H5.09^ in Gα_12_ interact with Q264^5.71^ in G_12_D. Screening of mutations at these positions in G_12_D identified G_12_D mutants that enhanced coupling with Gα_12_ and to an even greater extent with Gα_13_. Combined mutations, most notably the dual Y176^34.53^H and Q264^5.71^R mutant, further enhanced Gα_12__/__13_ coupling, thereby serving as a potential Gα_12/13_-DREADD. Such novel Gα_12/13_-DREADD may be useful in future efforts to develop drugs that target Gα_12/13_ signaling as well as to identify their therapeutic indications.

## Introduction

G-protein-coupled receptors (GPCRs) are the largest superfamily of membrane proteins and are involved in virtually all physiological phenomena^1,2^. GPCRs regulate intracellular pathways by binding extracellular ligands and transmitting signals across the plasma membrane to intracellular effector molecules via heterotrimeric G proteins composed of Gα, Gβ, and Gγ subunits^3^. The 16 Gα subtypes in human cells are classified into four Gα subfamilies (Gα_s_, Gα_i_, Gα_q_, and Gα_12_) that transmit unique signals (e.g., cAMP, Ca^2+^, inositol phosphate, and Rho activation) and Gα subtypes belonging to each of these subfamilies have been thought to exert similar functions^1^. However, recent studies have revealed that subtypes belonging to the same subfamily have different patterns of coupling to GPCRs and exert distinct functions, requiring further analysis focusing on each subtype^4–6^.

Gα_12_ and Gα_13_, which belong to the G_12_ subfamily, activate the small GTPase Rho through Rho-specific guanine nucleotide exchange factors^7^. Some reports indicate that Gα_12_ and Gα_13_ are functionally different, interacting with different effector target proteins leading to different outcomes. For example, Gα_13_-knockout mice are embryonic lethal due to defective angiogenesis, whereas Gα_12_ knockout mice develop normally despite the expression of both subtypes in the vascular endothelial cells^8–10^. This phenotypic difference has been attributed to myocyte-specific enhancer factor-2 (MEF2), which is specifically activated downstream of Gα_13_^11^. Several other effector proteins, such as Hsp90 and PYK2, have been identified to bind specifically to Gα_12_ or Gα_13_, respectively^12,13^.

Some GPCRs selectively couple to either Gα_12_ or Gα_13_, indicating that they are used effectively in various physiological conditions. In our previous study, we generated a dataset quantifying the coupling of 11 chimeric Gα proteins including Gα_12_ and Gα_13_ to 148 GPCRs by using TGFα shedding assay^5^. A meta-analysis integrating this dataset with the Guide to Pharmacology and the Bouvier datasets showed that 25 GPCRs were found to be coupled to both Gα_12_ and Gα_13_, 5 GPCRs specifically to Gα_12_, and 8 GPCRs specifically to Gα_13_^5,14–16^. However, it remains uncertain how GPCRs interact with Gα_12_ or Gα_13_ and what determines their coupling selectivity.

The C-terminal helix (α5 helix) of the Gα subunits is an important determinant for coupling selectivity. The α5 helix consists of 26 amino acids and interacts with the transmembrane core of GPCRs, occupying > 70% of the GPCR–G protein interface. Structural studies of the GPCR–Gα protein complex and cell-based assays confirmed the role of the α5 helix in determining Gα coupling selectivity^17–19^. In GPR35, out of the eight amino acids of the α5 helix that differ between Gα_12_ and Gα_13_, G.H5.23, the fourth residue from the C-terminus of the Gα subunit, contributes to its Gα_13_ preference over Gα_12_^20^. Thus, focusing on the interaction between a receptor and the α5 helix would help determine the key elements regulating the coupling selectivity between Gα_12_ and Gα_13_.

We have previously generated a Gα_12_-coupled Designer Receptor Exclusively Activated by Designer Drugs (DREADD) through a series of modifications. The first generation of Gα_12_-coupled DREADD was designed by modifying Gα_q_-coupled DREADD (M3D) based on the TGFα shedding assay database^5,21^. Subsequently, an F93^1.57^V mutation was introduced into the first-generation DREADD to create a second-generation Gα_12_-DREADD (G_12_D), which couples strongly to Gα_12_ but weakly to Gα_13_^22^. In the present study, we used the second generation G_12_D as an experimental model for analyzing the mechanism and determinants of Gα_12_-vs-Gα_13_-coupling selectivity.

In this study, by utilizing chimeric Gα proteins, we identified the specific residues in the α5 helix of Gα_12_ responsible for Gα_12_ selectivity. Through molecular dynamics analysis, we pinpointed residues in G_12_D that involve interactions with the selectivity determinants in the α5 helix. Finally, by mutating G_12_D, we successfully developed a Gα_12/13_-DREADD that increased both Gα_12/13_ coupling to similar extents.

## Results

### The Gα_12_ selectivity of G_12_D requires specific amino acid residues in the α5 helix including isoleucine G.H5.23

**Figure 1:**
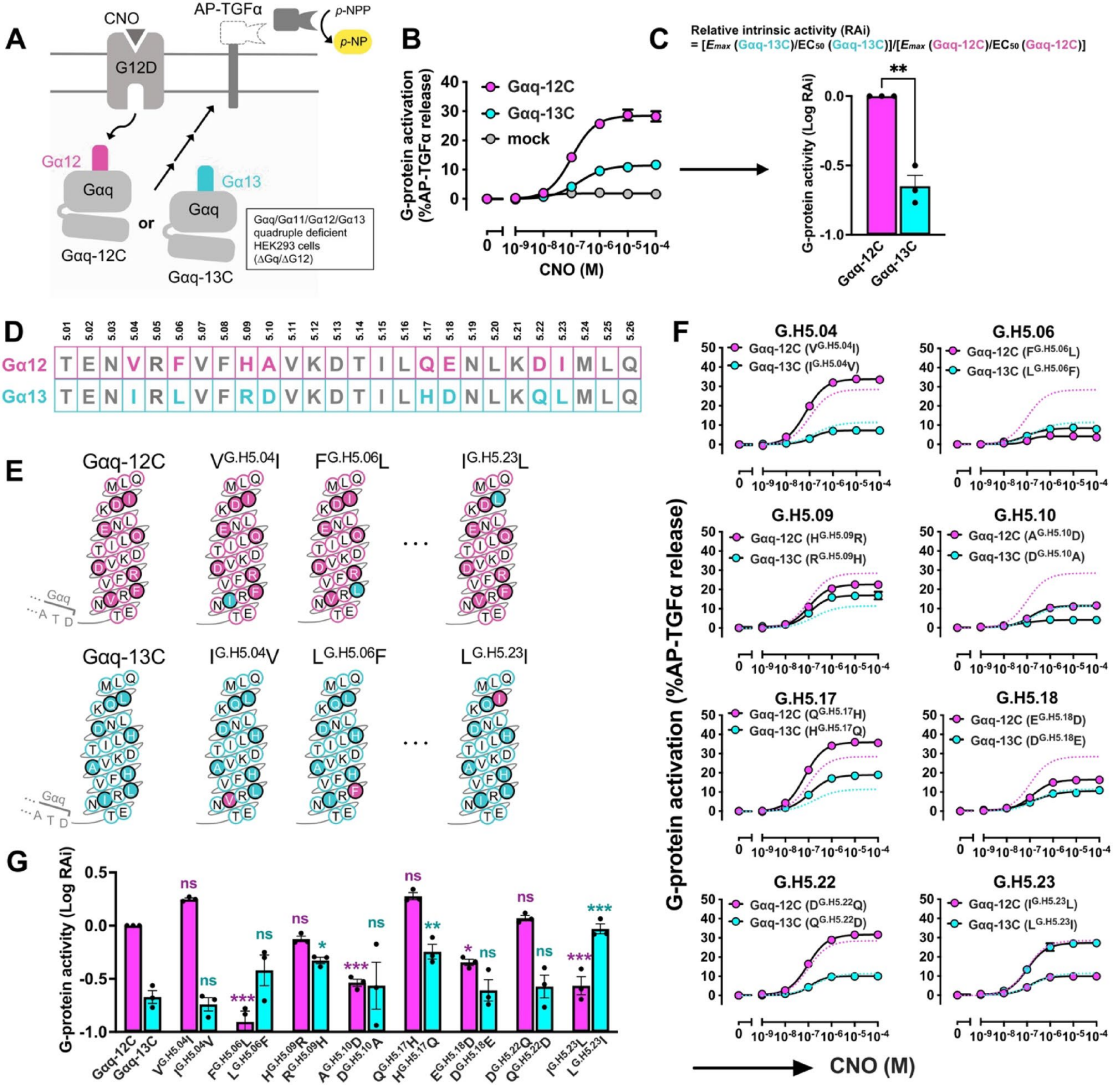
Identification of residues in the α5 helix involved in the Gα_12_-vs-Gα_13_-coupling selectivity by the TGFα shedding assay. (**A**) Schematic representation of the TGFα shedding assay for evaluating coupling between G_12_D and Gα_q-12C_ or Gα_q-13C_. G_12_D, chimeric Gα (Gα_q-12C_ or Gα_q-13C_), and AP-TGFα are transiently expressed in ∆G_q_/∆G_12_ cells. Upon stimulation with CNO, G_12_D is coupled with the chimeric Gα, of which activation leads to ectodomain shedding of the AP-TGFα reporter. Cleaved AP-TGFα is quantified by measuring AP activity in the conditioned media based on the production of para-nitrophenol (*p*-NP) from para-nitrophenyl phosphate (*p*-NPP). See the method section for detail. (**B**) Concentration–response curve for the TGFα shedding responses induced by G_12_D activation upon CNO stimulation. The vehicle-treated condition is set as the baseline. The magenta and cyan circles represent the response under conditions expressing Gα_q-12C_ and Gα_q-13C_, respectively. (**C**) Logarithmic values of relative intrinsic activity (RAi) calculated from (B) using the formula shown in the figure. The values were normalized by the Gα_q-12C_ expressing condition. Bars and error bars represent the mean and SEM, respectively, for three independent experiments with each dot representing an individual experiment. ** represents *P* < 0.01 with the two-tailed *t*-test. (**D**) Amino acid sequence of the α5 helices of Gα_12_ and Gα_13_. Positions where amino acids differ between Gα_12_ and Gα_13_ are highlighted in magenta and cyan, respectively. (**E**) Design of swapped mutants in the Gα_q-12C_ and the Gα_q-13C_ backbones. Mutants in positions G.H5.09, G.H5.10, G.H5.17, G.H5.18, and G.H5.22 are omitted. (**F**) Concentration–response curve for the TGFα shedding responses of the swapped mutants. The dashed lines represent the responses of Gα_q-12C_ (magenta) and Gα_q-13C_ (cyan) shown in (B). In all panels, the symbols and error bars represent the mean and SEM, respectively, for three independent experiments performed in triplicate. For many data points, the error bars are smaller than the symbols and, thus, are not visible. (**G**) Logarithmic values of RAi of the swapped mutants calculated from (F). Bars and error bars represent the mean and SEM, respectively, for three independent experiments with each dot representing an individual experiment. ns, *, ** and *** represent *P* > 0.05, < 0.05, < 0.01 and < 0.001, respectively, with two-way ANOVA, followed by the Dunnett’s multiple comparisons test (Gα_q__-12_ mutants with wild-type Gα_q-12C_; Gα_q__-13_ mutants with wild-type Gα_q-13C_).

To determine which amino acid residues in the α5 helix are critical for preferential coupling for Gα_12_ over Gα_13_ by G_12_D, we constructed chimeric Gα proteins, in which the α5 helix (G.H5.01–G.H5.26) of Gα_q_ was replaced by the α5 helix of Gα_12_ or Gα_13_ (Gα_q-12C_ or Gα_q-13C_, respectively). By following a previous strategy^5^ where we introduced a chimeric Gα subunit in the Gα_q_/Gα_11_/Gα_12_/Gα_13_ quadruple-deficient HEK293 cells (∆G_q_/∆G_12_ cells), we herein co-expressed Gα_q-12C_ or Gα_q-13C_ along with G_12_D and a reporter gene encoding alkaline phosphatase-tagged TGFα (AP-TGFα) in ∆Gq/∆G_12_ cells. Cells were stimulated with increasing concentrations of clozapine-N-oxide (CNO) and the relative coupling of Gα_q-12C_ and Gα_q-13C_ to G_12_D was evaluated by measuring the activity of cleaved AP-TGFα (Fig. 1A,B). The relative intrinsic activities (RAi) of Gα_q-13C_ to Gα_q-12C_ was calculated from the concentration–response curves in the TGFα shedding assay^5,23^. This parameter analysis showed that the RAi was 4.6-fold higher in cells expressing Gα_q-12C_ than in cells expressing Gα_q-13C_, confirming a previous report that G_12_D preferentially couples with Gα_q-12C_^5^ (Fig. 1C). The amino acid sequences of the α5 helices of Gα_12_ and Gα_13_ differ at the following positions: G.H5.04, G.H5.06, G.H5.09, G.H5.10, G.H5.17, G.H5.18, G.H5.22, and G.H5.23 (Fig. 1D). To determine which of these amino acid differences confer coupling selectivity, we constructed eight pairs of chimeric Gα protein mutants. Specifically, the amino acid at one of the eight variable positions in the α5 helix was switched from its Gα_12_ residue to its Gα_13_ residue or vice versa, while the rest of the Gα_q-12C_ and Gα_q-13C_ protein sequences remained unchanged (Fig. 1E, Supplementary Fig. 1). We found that coupling selectivity was reversed when the amino acid at the position G.H5.23 was reversed; thus, Gα_q-13C_ carrying a leucine to isoleucine mutation (*i.e.*, Gα_q-13C_ [L^G.H5.23^I]) coupled preferentially to G_12_D, while the reverse mutants, Gα_q-12C_ (I^G.H5.23^L), did not (Fig. 1F,G, Supplementary Fig. 2). In addition, Gα_q-13C_ (R^G.H5.09^H) enhanced coupling with G_12_D, while Gα_q-12C_ (H^G.H5.09^R) tended to decrease it (Fig. 1F,G, Supplementary Fig. 2). These results indicate that Gα_12_ and Gα_13_ coupling specificity is attributable to the specific residues in the α5 helix, most notably at the position G.H5.23 (isoleucine and leucine, respectively).

### MD simulations reveal interactions involving Gα_12_ selectivity of G_12_D

**Figure 2:**
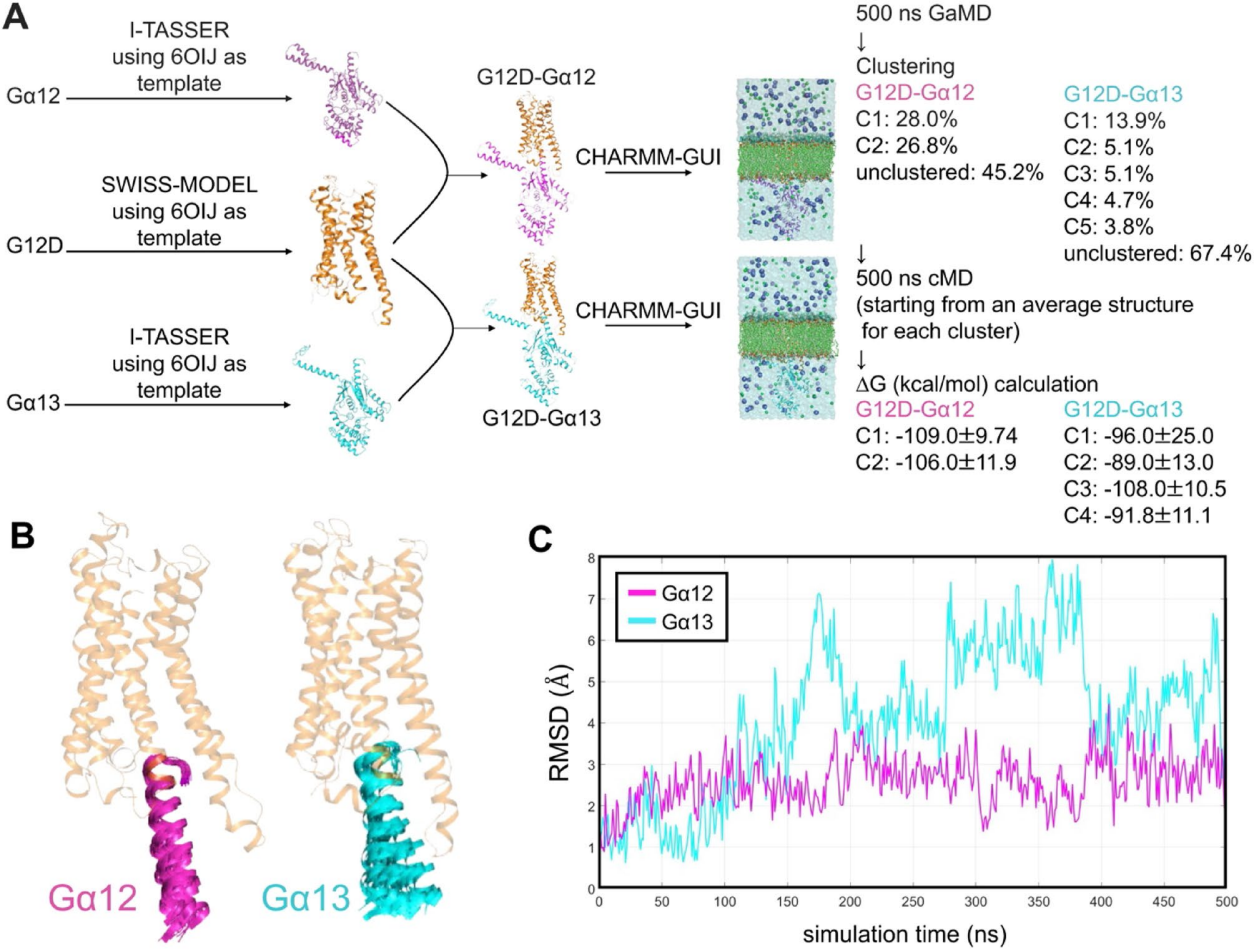
Comparison of the stability between the G_12_D–Gα_12_ and the G_12_D–Gα_13_ complexes through MD simulations. (**A**) Schematic representation of the molecular dynamics simulations. On the right side, the occupancy (%) of each cluster and the ∆G (kcal/mol) calculated from the cMD results are displayed. (**B**) Overlap of snapshots every 20 ns from 500-ns cMD simulations starting from each cluster 1 (C1). For clarity, G_12_D is shown with the average structure of each simulation and residues other than the α5 helix are hidden. (**C**) RMSD for the α5 helix calculated from 500-ns cMD simulations starting from C1.

To gain insight into the Gα_12_ selectivity of G_12_D, we performed molecular dynamics (MD) simulations of the G_12_D–Gα_12_ and the G_12_D–Gα_13_ complexes. The two complexes were modeled with SWISS-MODEL and I-TASSER structure prediction software using M1R–Gα_11_ complex structure (PDB: 6OIJ) as a template and embedded into lipid bilayers using CHARMM-GUI^24–27^ (Fig. 2A). Both models were simulated for 500 ns using a conventional MD protocol to estimate the Poisson-Boltzmann-based energy of the Gα protein to the receptor as described previously^28^. Because calculations of the G_12_D–Gα_12_ total energy indicated that low energy states were not conformationally sampled, 500-ns Gaussian Accelerated Molecular Dynamics (GaMD) simulations were performed^29^. The GaMD snapshots of the G_12_D–Gα_12_ complex clustered into two distinct conformations and the snapshots of the G_12_D–Gα_13_ complex clustered into five distinct conformations, indicating that the latter complex was more dynamic. The average structure that best represented each cluster was then chosen as the starting point for a final 500-ns conventional MD allowing for unbiased free energy calculations and receptor–G protein interaction analyses. For the G_12_D–Gα_12_ complex, most structural variation between the two clusters was in the internal dynamics of the Gα_12_ protein and TM6 of the receptor. For the G_12_D–Gα_13_ complex, most structural variation among the five clusters was in the internal dynamics of the Gα_13_ protein, the positioning of its α5 helix, and the intracellular facing regions of TM5/TM6 domains of the receptor.

The results of the MD simulations revealed significant differences in the stability of Gα_12_ and Gα_13_ interaction with G_12_D. From the MD trajectories, we calculated the total energy (∆G) of Gα_12_ and Gα_13_ through MMPBSA analysis^30^. We found that ∆G was weaker (less negative) in the Gα_13_ simulation than in the Gα_12_ simulation, indicating that the interaction between G_12_D and Gα_13_ is less stable than that between G_12_D and Gα_12_ (Fig. 2A). We also observed that the position in the α5 helix of Gα_13_ in the transmembrane core of G_12_D was variable, while its position in Gα_12_ was more stable and essentially stayed in the transmembrane core of G_12_D (Fig. 2B). Indeed, the root-mean-square deviation (RMSD) for the α5 helix was higher in the Gα_13_ simulation than in the Gα_12_ simulation for the most populated cluster (C1; Fig. 2C). RMSD analysis for the α5 helix for all clusters showed that, compared with the C1 cluster of the G_12_D–Gα_12_ complex, all of the other clusters including those of the G_12_D–Gα_13_ complex were sampled more frequently in positions with RMSD scores beyond 4 Å (Supplementary Fig. 3A). These observations are consistent with the results of cell-based assays (Fig. 1F,G).

**Figure 3:**
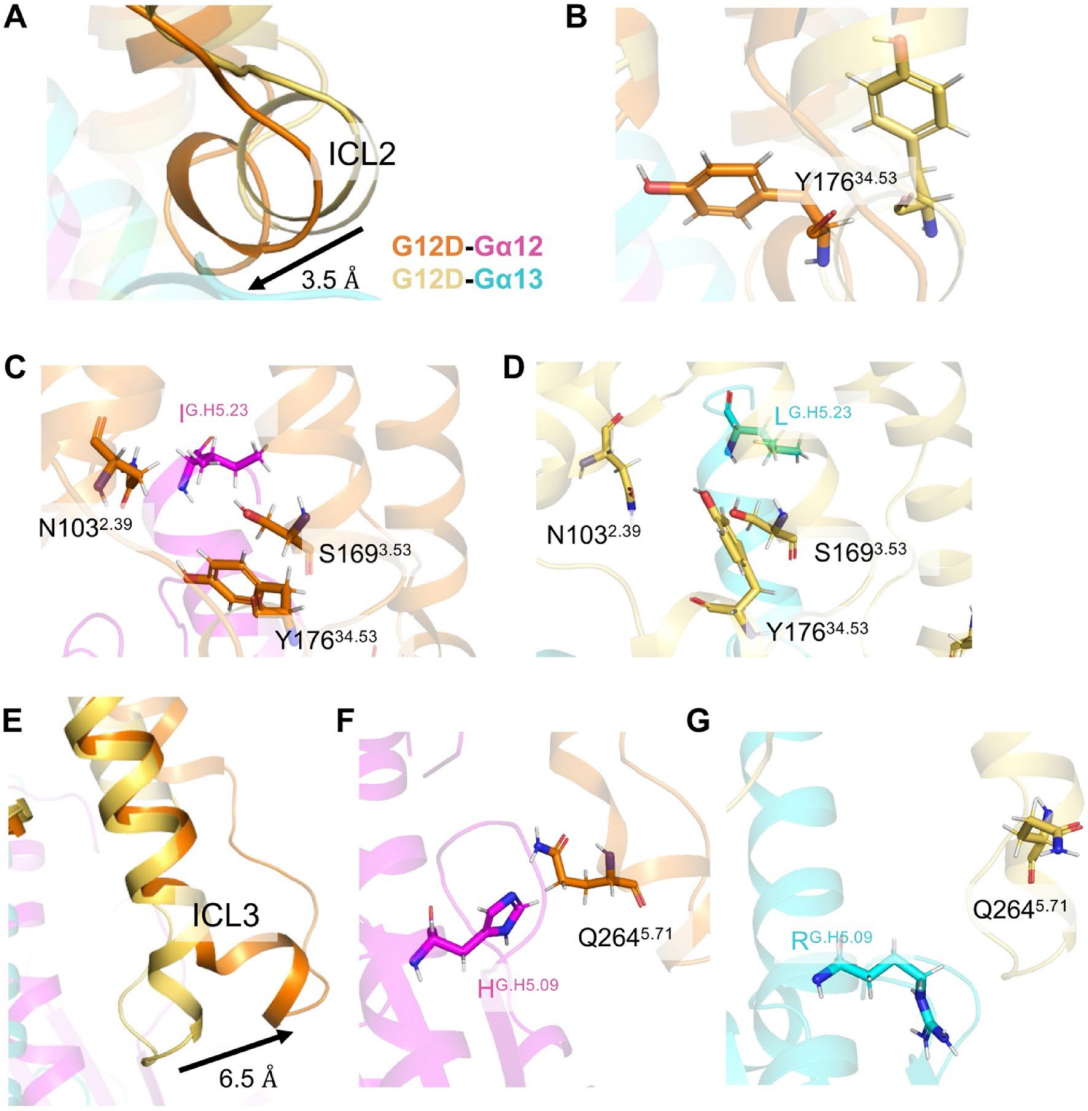
Structural comparison of the G_12_D–Gα_12_ and the G_12_D–Gα_13_ complexes through MD simulations. (**A**) A comparison of ICL2 structures between the G_12_D–Gα_12_ and the G_12_D–Gα_13_ complexes. Shown are representative structures during the 500-ns cMD simulations of the G_12_D complexes, each started from the most populated clusters (C1) observed in the GaMD simulations. These structures were aligned with G_12_D transmembrane bundles. In the G_12_D–Gα_12_ complex, G_12_D and Gα_12_ are shown in orange and magenta, respectively. In the G_12_D–Gα_13_ complex, G_12_D and Gα_12_ are shown in yellow and cyan, respectively. (**B**) A comparison of the positions of Y176^34.53^ between the G_12_D–Gα_12_ and the G_12_D–Gα_13_ complexes. (**C**,**D**) Detailed positions of Gα_12_ Ile^G.H5.23^ (C) and Gα_13_ Leu^G.H5.23^ (D) in the transmembrane core of G_12_D. (**E**) A comparison of ICL3 structures between the G_12_D–Gα_12_ and the G_12_D–Gα_13_ complexes. (**F**,**G**) Detailed orientations of G.H5.09 residue.

The most notable differences in the simulations of the G_12_D–Gα_12_ and the G_12_D–Gα_13_ complexes were in ICL2. More specifically, when comparing the average structures of the two complexes, the position of ICL2 was tilted 3.5 Å more toward the α5 helix in the G_12_D–Gα_12_ complex than in the G_12_D–Gα_13_ complex (Fig. 3A). Among residues in the ICL2, Y176^34.53^ showed the most noticeable difference between the simulations (Fig. 3B). In the simulation of the G_12_D–Gα_12_ complex, Y176^34.53^ was positioned between N103^2.39^ and S169^3.53^ and oriented toward Gα_12_ residue I^G.H5.23^, such that Y176^34.53^, N103^2.39^, and S169^3.53^ form a pocket for the methyl group of I^G.H5.23^ (Fig. 3B,C, Supplementary Fig. 3B). These observations show that the transmembrane core of G_12_D contains a pocket that accommodates I^G.H5.23^, and this likely stabilizes the G_12_D–Gα_12_ complex in a low energy state. On the other hand, in the α5 helix of Gα_13_, L^G.H5.23^ appeared disoriented and tilted toward TM5 and TM6 (Fig. 3D), while G_12_D Y176^34.53^ was oriented upward and failed to form a pocket with N103^2.39^ and S169^3.53^, likely disfavoring an interaction between these residues and Gα_13_ L^G.H5.23^ (Fig. 3D, Supplementary Fig. 3B). Together, these observations suggest that the pocket formed by G_12_D residues N103^2.39^, S169^3.53^ and Y176^34.53^ plays a significant role in the preferential coupling between G_12_D and Gα_12_.

In the G_12_D structures, we found another difference in the region composed of the C-terminal portion of the TM5, ICL3 and the N-terminal portion of TM6 (TM5–ICL3–TM6). In the simulation of the G_12_D–Gα_12_ complex, the TM5–ICL3–TM6 region was located farther from the transmembrane core side than in the Gα_13_ simulation (Fig. 3E). In general, the active form of GPCR displays an outward shift of TM5–ICL3–TM6, which facilitates the accommodation of α5 helix^17^. Therefore, the TM5–ICL3–TM6 opening observed in the G_12_D–Gα_12_ complex likely reflects a favorable form for coupling. In the simulations, TM5–ICL3–TM6 was in the proximity of the G.H5.09 residue position out of the eight different residues in the α5 helices of Gα_12_ and Gα_13_. Consistent with the observations, in the mutant experiment in Fig. 1E,F, the mutants in which these positions of Gα_13_ were swapped for Gα_12_ residues showed enhanced coupling to G_12_D, indicating their involvement in the selectivity. In the simulations, the G.H5.09 residue appeared to be closest to Q264^5.71^ in G_12_D (Fig. 3F,G). Therefore, Q264^5.71^, in addition to the previously mentioned N103^2.39^, S169^3.53^ and Y176^34.53^, are potential residues involved in the selectivity.

### N103^2.39^, S169^3.53^, Y176^34.53^ and Q264^5.71^ in G_12_D collectively contribute to the Gα_12_-vs-Gα_13_ selectivity

**Figure 4:**
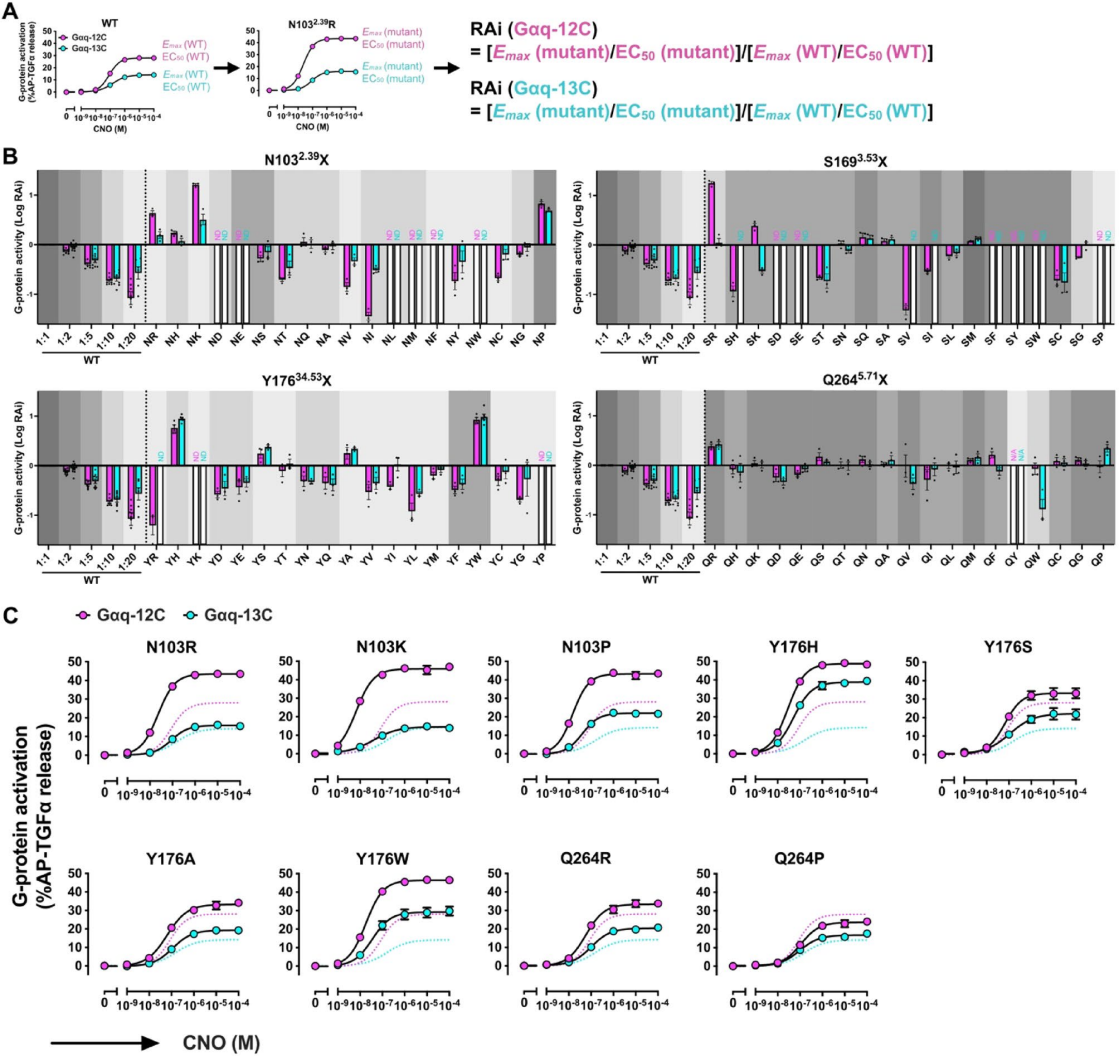
Determining G_12_D residues involved in the Gα_12_-vs-Gα_13_ selectivity. (**A**) Calculation method for RAi of the G_12_D mutants. (**B**) Logarithmic values of RAi of the G_12_D mutants. ∆G_q_/∆G_12_ cells transiently expressing the indicated G_12_D mutants along with AP-TGFα and Gα_q-12C_ (or Gα_q-13C_) (Gα_q-12C_-expressing condition and Gα_q-13C_-expressing condition are shown as magenta and cyan, respectively). The cell-surface expression levels are shown in gray with different intensities to allow comparison with the wild-type G_12_D. WT (1:2) to (1:20) denote twofold to 20-fold less volumes, respectively, of transfected plasmids than those of the mutant plasmids. Bars and error bars represent the mean and SEM, respectively, for 3–15 independent experiments with each dot representing an individual experiment. The data for WT and its dilutions are reused in all panels. (**C**) Concentration–response curve for the TGFα shedding responses of the representative G_12_D mutants. The dashed lines represent the responses of the wild-type G_12_D. In all panels, the symbols and error bars represent the mean and SEM, respectively, for three independent experiments. For many data points, the error bars are smaller than the symbols and, thus, are not visible.

To examine the importance N103^2.39^, S169^3.53^, Y176^34.53^ and Q264^5.71^ in the Gα_12_-vs-Gα_13_-coupling selectivity as well as to generate a G_12_D mutant with enhanced Gα_13_ coupling, we next performed mutation studies in G_12_D. We engineered 19 amino acid substitution mutations at these positions and examined the coupling of Gα_12_ and Gα_13_ for a total of 76 mutants. Each G_12_D mutant carried an N-terminal FLAG-epitope tag to allow cell surface expression to be quantified by flow cytometry. Most mutants carrying substitutions of N103^2.39^ and Y176^34.53^ were expressed at a lower level than the wild-type (WT) control, while expression of mutants carrying substitutions of S169^3.53^ and Q264^5.71^ showed relatively smaller changes in expression level (Supplementary Fig. 4). Next, the Gα_12_-vs-Gα_13_ selectivity of these mutants was assessed using the TGFα shedding assay. The RAi values were calculated using the equation shown in Fig. 4A, offering indexes into the extent to which the mutants enhanced (or reduced) coupling to Gα_q-12C_ and Gα_q-13C_ relative to the wild-type G_12_D (Fig. 4B,C, Supplementary Fig. 5–9). G_12_D mutants — with substitutions at N103^2.39^ with R, K or P; Y176^34.53^ with H, S, A or W; or Q264^5.71^ with R or P — showed higher RAi and stronger coupling to Gα_q-12C_ and Gα_q-13C_ than WT. In particular, Y176^34.53^H and Y176^34.53^W greatly enhanced coupling to Gα_q-13C_, and this enhancement effect was stronger than that for Gα_q-12C_. None of the S169^3.53^ mutations enhanced coupling to Gα_q-13C_, whereas S169^3.53^R and S169^3.53^K selectively enhanced coupling to Gα_q-12C_. Although these mutants of G_12_D display varying degrees of selective coupling to Gα_12_ or Gα_13_, the results of these experiments confirm the importance of N103^2.39^, S169^3.53^, Y176^34.53^, Q264^5.71^ as determinants of G_12_D coupling.

**Figure 5:**
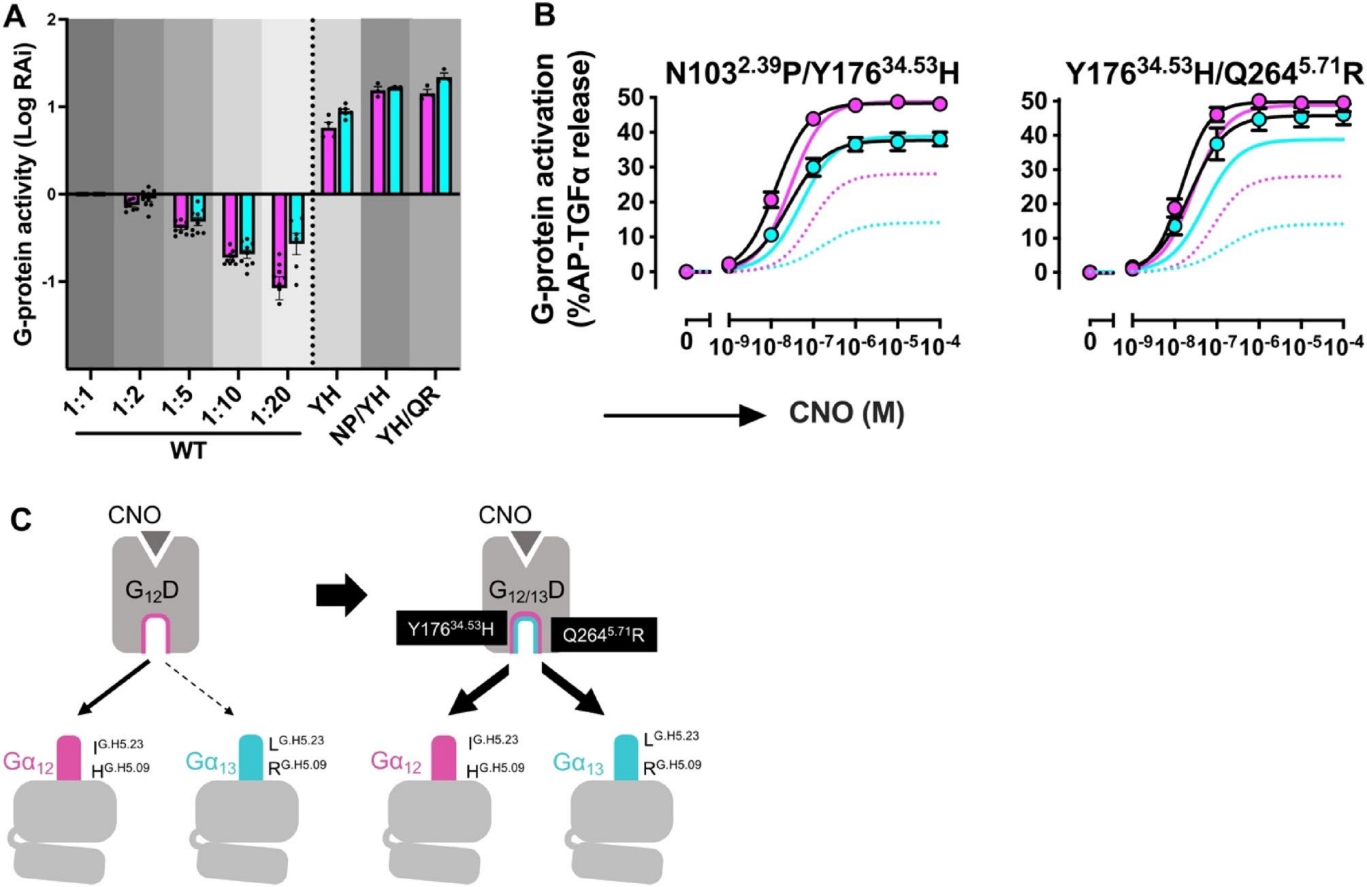
Development of Gα_12/13_-DREADD. (**A**) Logarithmic values of RAi of the G_12_D mutants (Gα_q-12C_ expressing condition = magenta, Gα_q-13C_ expressing condition = cyan). The cell-surface expression levels of the mutants are shown in gray with different intensities to allow comparison with the wild-type G_12_D. WT (1:2) to (1:20) denote twofold to 20-fold less volumes of transfected plasmids, respectively, than those of the mutant plasmids. Bars and error bars represent the mean and SEM, respectively, for three independent experiments performed in triplicate. The data for WT and its dilutions are reused from Fig. 4. (**B**) Concentration–response curve for the TGFα shedding responses of the representative G_12_D mutants. The dashed lines and solid lines represent the responses of the wild-type G_12_D and Y176^34.53^H, respectively. In all panels, the symbols and error bars represent the mean and SEM, respectively, for three independent experiments performed in triplicate. For many data points, the error bars are smaller than the symbols and, thus, are not visible. (**C**) A schematic model of the Y176^34.53^H/Q264^5.71^R mutant showing potent coupling to both Gα_12_ and Gα_13_ via recognition of the three residues in the α5 helix.

To further enhance coupling between Gα_13_ and G_12_D, we introduced additional mutations on top of the Y176^34.53^H mutant, which demonstrated the highest Gα_13_-coupling capability in both RAi and *E*_*max*_ parameters (Fig. 4B,C, Supplementary Fig. 5–9). We chose N103^2.39^P and Q264^5.71^R because they enhance coupling to Gα_q-13C_ (Fig. 4B,C, Supplementary Fig. 5–9). Compared to Y176^34.53^H, both N103^2.39^R/Y176^34.51^H and Y176^34.53^H/Q264^5.71^R showed increased RAi values under both Gα_q-12C_ and Gα_q-13C_ expressing conditions. Furthermore, Y176^34.53^H/Q264^5.71^R also exhibited an increasing effect on *E*_*max*_ (Fig. 5A,B, Supplementary Fig. 10). Based on these results, we designated Y176^34.53^H/Q264^5.71^R double mutant, which exhibits strong coupling to the α5 helices of Gα_12_ and Gα_13_, as Gα_12/13_-DREADD (Fig. 5C).

## Discussion

In this study, we showed that I^G.H5.23^ at position G.H5.23 in the α5 helix of Gα_12_ is a major determinant of selective coupling between Gα_12_ and G_12_D. Conversely, L^G.H5.23^ in Gα_13_ has been reported to contribute to the Gα_13_ selectivity of GPR35^20^. These findings support the notion that the interaction between G.H5.23 in the α5 helix and target GPCRs is critical for the Gα_12_-vs-Gα_13_ coupling selectivity. Structural analyses of S1PR2–Gα_13_ and GPR35–Gα_13_ complexes have revealed that L ^G.H5.23^ is oriented toward TM2 and TM3 and is accommodated by a pocket formed by residues 2.39, 3.53, and 34.53^31,32^. In particular, in the S1PR2–Gα_13_ complex, ICL2 lies inside the transmembrane core, highlighting the importance of 34.53^31^. These studies support the accuracy of our MD-simulated structures of G_12_D–Gα_12_ and G_12_D–Gα_13_ complexes. Notably, the molecular properties of isoleucine and leucine are very similar except for the different positions of the methyl groups in the amino acid side chains, and yet GPCRs distinguish one from the other. This is likely achieved by how well the pocket in the GPCR transmembrane core accommodates the isoleucine versus the leucine side chain. In the MD simulations described in the present study, the methyl group of Gα_13_ L^G.H5.23^ is predicted to encounter steric hindrance in the G_12_D pocket formed by TM2, TM3, and ICL2, which would create instability in the G_12_D–Gα_13_ complex. This idea is consistent with the fact that amino acid substitutions that altered the shape of the transmembrane core of G_12_D enhanced coupling to Gα_13_, presumably by creating a pocket that is optimized to accommodate L^G.H5.23^.

One caveat of this study is that it only identifies determinants of GPCR coupling selectivity in the 26-amino-acid α5 helix of Gα_12_ and Gα_13_. The rationale for this limitation is that prior studies using chimeric G proteins revealed a crucial role of the α5 helix, especially G.H5.23 and G.H5.24 positions, for determining coupling selectivity^18,19^. By focusing here on the α5 helix, we avoid difficulties arising from differences in the efficiency of the TGFα shedding assay as a surrogate measure of Gα_12_- and Gα_13_-mediated signaling. However, additional determinants of G protein coupling specificity exist in other protein regions and structures such as the αN–β1 hinge and the β2–β3 loop^33–35^. To fully understand the mechanisms underlying Gα_12_-vs-Gα_13_-coupling selectivity, a broad examination of determinants of coupling selectivity linked to these elements will be needed in the future. Another caveat is that the present study is based on structural models instead of experimentally verified protein structural data. That said, the full Gα subunit was used in the simulations to ensure that the α5 helix had the proper local environment for positioning relative to the receptor. In addition, to ensure the accuracy of the modeled structures, we performed GaMD followed by clustering before the production runs to ensure that we captured stable conformations of the receptor and the Gα proteins in the modeled complexes. The resulting Gα_12_ and Gα_13_ simulations showed significant differences in stability. In the future, once the structures of GPCR–Gα_12_ or GPCR–Gα_13_ complexes are resolved, they can be used to validate and/or improve upon the simulations presented here.

DREADDs that couple specifically to one or more Gα proteins were designed for and have been useful in drug discovery research^36^. Gα_q_-DREADD and Gα_i_-DREADD have been universally used to regulate neuronal activation^37,38^. DREADDs have also been used to analyze G protein signaling in a particular cell population and to predict the effects of agonists that activate specific G-protein-signaling pathways. Administration of CNO to mice genetically modified to express DREADD in a cell type-specific fashion allows for spatio-temporal regulation of specific G-protein signaling. For instance, analyses of DREADD-transgenic mice have revealed the enhancing effect of hepatic glucose production by Gα_s_, Gα_i_, and Gα_q_ signaling in hepatocytes, and our G_12_D discovered the enhancing effect of browning by Gα_12_ signaling in adipocytes^22,39–41^. In the present study, an engineered mutant of G_12_D created the novel Gα_12/13_-DREADD, which is useful for research on Gα_12/13_ signaling. In addition, mutations that enhance coupling to Gα_12_, including N103^2.39^R, N103^2.39^K, S169^3.53^R, and S169^3.53^K, were discovered in the present study. These mutations could be used to design next-generation G_12_D that are more potent for Gα_12_. Gα_12_ and Gα_13_ signaling have been reported to have important functions in the immune system. Gα_12/13_-coupled GPCRs such as S1PR2 activate Gα_12/13_ signaling and regulate immune cell activities and functions in a temporally regulated manner^42,43^. Immune cell species-specific expression of Gα_12/13_-DREADD and administration of CNO at various stages of the immune response will aid in a comprehensive understanding of the tightly regulated Gα_12/13_ signaling function. However, for functional analysis of Gα_13_ signaling alone, future modification of Gα_12/13_-DREADD will be necessary to reduce its coupling to Gα_12_.

## Methods

### Reagents and plasmids

CNO dihydrochloride was purchased from Tocris Bioscience or synthesized at the International Institute for Integrative Sleep Medicine (WPIIIIS), University of Tsukuba. The detailed synthetic method was described previously^22^. A plasmid encoding alkaline phosphatase (AP)-TGFα was described previously^5^. G_12_D (M3D-GPR183/ICL3 F93^1.57^V) and G_12_D mutants carrying the N-terminal FLAG-epitope tag (DYKDDDDK) were cloned into the pcDNA3.1 expression vector^5^. The amino-acid sequence of FLAG-epitope-tagged G_12_D is denoted below; the ICL3 swap, the F93^1.57^V mutation, the FLAG-epitope tag and the substitutions of Y^3.33^C and A^5.46^G mutations from the human M3 receptor are underlined and highlighted in bold:

MDYKDDDDKGSTLHNNSTTSPLFPNISSSWIHSPSDAGLPPGTVTHFGSYNVSRAAGNFSSPDGTTDDPLGGHTVWQVVFIAFLTGILALVTIIGNILVIVS**V**KVNKQLKTVNNYFLLSLACADLIIGVISMNLFTTYIIMNRWALGNLACDLWLAID**C**VASNASVMNLLVISFDRYFSITRPLTYRAKRTTKRAGVMIGLAWVISFVLWAPAILFWQYFVGKRTVPPGECFIQFLSEPTITFGTAIAG**F**YMPVTIMTILYWRIYKETE**RTAKQNPLTEKSGVE**KKAAQTLSAILLAFIITWTPYNIMVLVNTFCDSCIPKTFWNLGYWLCYINSTVNPVCYALCNKTFRTTFKMLLLCQCDKKKRRKQQYQQRQSVIFHKRAPEQAL.

Gα_q-12C_, Gα_q-13C_, and their mutants were inserted into the pcDNA3.1 expression vector. The amino acid sequence of Gα_q-12C_ and Gα_q-13C_ are as follows (underlining denotes the α5 helices of Gα_12_ and Gα_13_).

Gα_q-12C_:

MTLESIMACCLSEEAKEARRINDEIERQLRRDKRDARRELKLLLLGTGESGKSTFIKQMRIIHGSGYSDEDKRGFTKLVYQNIFTAMQAMIRAMDTLKIPYKYEHNKAHAQLVREVDVEKVSAFENPYVDAIKSLWNDPGIQECYDRRREYQLSDSTKYYLNDLDRVADPAYLPTQQDVLRVRVPTTGIIEYPFDLQSVIFRMVDVGGQRSERRKWIHCFENVTSIMFLVALSEYDQVLVESDNENRMEESKALFRTIITYPWFQNSSVILFLNKKDLLEEKIMYSHLVDYFPEYDGPQRDAQAAREFILKMFVDLNPDSDKIIYSHFTCATDTENVRFVFHAVKDTILQENLKDIMLQ;

Gα_q-13C_:

MTLESIMACCLSEEAKEARRINDEIERQLRRDKRDARRELKLLLLGTGESGKSTFIKQMRIIHGSGYSDEDKRGFTKLVYQNIFTAMQAMIRAMDTLKIPYKYEHNKAHAQLVREVDVEKVSAFENPYVDAIKSLWNDPGIQECYDRRREYQLSDSTKYYLNDLDRVADPAYLPTQQDVLRVRVPTTGIIEYPFDLQSVIFRMVDVGGQRSERRKWIHCFENVTSIMFLVALSEYDQVLVESDNENRMEESKALFRTIITYPWFQNSSVILFLNKKDLLEEKIMYSHLVDYFPEYDGPQRDAQAAREFILKMFVDLNPDSDKIIYSHFTCATDTENIRLVFRDVKDTILHDNLKQLMLQ.

### Cell culture and transfection

Parent and Gα_q_/Gα_11_/Gα_12_/Gα_13_ quadruple-deficient HEK293 cells^5^ were maintained in Dulbecco’s modified Eagle medium (DMEM, Nissui Pharmaceutical) supplemented with 10% fetal bovine serum (Gibco), 100 U/mL penicillin (Sigma-Aldrich), and 100 µg/mL streptomycin (Gibco) (complete DMEM) in a 37 °C incubator with 5% CO_2._ Transfection of plasmid DNAs was performed using lipofection reagent polyethylenimine solution (PEI Max, Polysciences). Typically, cells were seeded in each well of a 12-well culture plate at a cell density of 2 × 10^5^ to 3 × 10^5^ cells/mL in 1 mL complete DMEM and cultured for 1 day in a 37 °C incubator with 5% CO_2_. For transfection, plasmid solution was diluted in 50 µL Opti-MEM (Gibco) and mixed with 2.5 µL of 1 mg/mL PEI solution in 50 µL Opti-MEM. Cells were incubated for 1 day after transfection before use in an experiment.

### TGFα shedding assay

The TGFα shedding assay was performed as described previously^5,23^. Plasmid transfection was performed in a 12-well plate using a mixture containing 250 ng of plasmid DNA encoding AP-TGFα, 100 ng of plasmid encoding﻿ FLAG-G_12_D, and 50 ng of plasmid encoding Gα_q-12C_ or Gα_q-13C_. After incubation for 1 day, transfected cells were harvested by trypsinization, pelleted by centrifugation at 190 × *g* for 5 min, and suspended in 3.5 mL Hank’s Balanced Salt Solution (HBSS) containing 5 mM HEPES (pH 7.4). After incubation for 15 min at room temperature, during which spontaneous AP-TGFα shedding settled down, the cells were centrifuged at 190 × *g* for 5 min, and cell pellets were resuspended in 3.5 mL HBSS. The resuspended cells were plated in a 96-well plate at 90 µl per well (typically 24 total wells; triplicate measurement) and placed in a 37 °C incubator with 5% CO_2_ for 30 min. After incubation, 10 µl of 10× CNO serial dilution in HBSS containing 5 mM HEPES (pH 7.4) and 0.01% (v/w) bovine serum albumin (BSA) was added to each well and incubated for 1 h at 37 °C in 5% CO_2_. The 96-well cell plates were centrifuged at 190 × *g* for 2 min. After centrifugation, 80 µL supernatant from each well was transferred to another 96-well plate (conditioned media plate or CM plate), leaving attached cells and 20 µL supernatant in the original well of the cell plate. Then, 80 µL of para-nitrophenyl phosphate (*p*-NPP) solution (10 mM *p*-NPP; 40 mM Tris-HCl, pH 9.5; 40 mM NaCl; 10 mM MgCl_2_) was added to each well of each plate. Absorbance at 405 nm (Abs_405_) was measured for both plates before (background) and after a 1-h or 2-h incubation at room temperature using a microplate reader (SpectraMax 340 PC384, Molecular Devices). The percent of AP-TGFα release was calculated using the following equation:

$${\text{AP}} \mbox{-} {\text{TGF}}\upalpha \, (\% {\text{ CM}}) = \, [\Delta {\text{Abs}}_{{{4}0{\text{5 CM}}}} /\left( {\Delta {\text{Abs}}_{{{4}0{\text{5 CM}}}} + \, \Delta {\text{Abs}}_{{{4}0{\text{5 cell}}}} } \right)\; \times \;{1}.{25}]\; \times \;{1}00$$

where CM denotes conditioned media.

Vehicle-treated AP-TGFα (%CM) was subtracted from CNO-stimulated AP-TGFα (%CM) and the resulting value (%AP-TGFα release) was used to represent a G-protein response.

### Flow cytometry

∆Gq/∆G_12_ cells were seeded in a 12-well culture plate at a concentration of 3 × 10^5^ cells per mL (1 mL per well) 1 day before transfection. Transfection was performed using 100 ng of plasmid encoding FLAG-G_12_D (or mutant FLAG-G_12_D), 250 ng plasmid encoding AP-TGFα and 50 ng plasmid encoding Gα_q-12C_ (or Gα_q-13C_). After incubation for 1 day, cells were collected by adding 100 μL 0.53 mM EDTA-containing D-PBS, followed by 100 μL of 5 mM HEPES (pH 7.4)-containing HBSS. The cell suspension was transferred to a 96-well V-bottom plate and fluorescently labeled using anti-FLAG-epitope tag monoclonal antibody (Clone 1E6, FujiFilm Wako Pure Chemical; 10 μg per mL) diluted in 2% goat serum, 2 mM EDTA in D-PBS (blocking buffer), followed by goat anti-mouse IgG secondary antibody conjugated with Alexa Fluor 488 (Thermo Fisher Scientific; 10 μg per mL). After washing with D-PBS, the cells were resuspended in 200 μL of 2 mM EDTA in D-PBS and filtered through a 40-μm filter. Single-cell fluorescence was quantified using an EC800 flow cytometer with a 488-nm laser (Sony). Live cells were gated with a forward scatter (‘FS-Peak-Lin’) cutoff of 390 and a gain of 1.7. Mean fluorescence intensity of all recorded events (approximately 20,000 cells per sample) was analyzed with FlowJo software (FlowJo) and used for statistical analysis.

### Western blot

∆Gα_q_/∆G_12_ cells were lysed in SDS-PAGE sample buffer (62.5 mM Tris–HCl [pH 6.8], 50 mM dithiothreitol, 2% SDS, 10% glycerol, and 4 M urea) containing 1 mM EDTA, 1 mM phenylmethylsulfonyl fluoride and 2 mM sodium orthovanadate. Lysates of an equal number of cells were separated using 8% or 12.5% SDS-polyacrylamide gel electrophoresis. Subsequently, the proteins were transferred to a nitrocellulose membrane (GE Healthcare). The membrane was blocked in 5% skim milk in blotting buffer (10 mM Tris–HCl (pH 7.4), 190 mM NaCl, and 0.05% Tween 20), and proteins were labeled and visualized by immunoblotting with appropriate primary and secondary antibodies. The primary antibody used in this study was an anti-G_q_ goat polyclonal antibody (Abcam, ab128060, D-6, lot GR108939-6, 1:2000 dilution). The secondary antibody was horseradish peroxidase (HRP)-conjugated anti-goat IgG (American Qualex, A201PS lot 7A0327H). The membrane was soaked in luminol reagent (100 mM Tris–HCl (pH 8.5), 50 mg per mL of Luminol Sodium Salt HG (FujiFilm Wako Pure Chemical), 0.2 mM *p*-Coumaric acid and 0.03% (v/v) of H_2_O_2_). Chemiluminescence imaging was performed and band intensities were measured using Amersham Imager 680 (Cytiva).

### Modeling the G_12_D–Gα_12_ and the G_12_D–Gα_13_ complexes

To build computational models of the G_12_D–Gα_12_ and the G_12_D–Gα_13_ complexes, we used SWISS-MODEL and I-TASSER structure prediction software packages with the muscarinic acetylcholine receptor 1 (M1R)–G_11_ protein complex (PDB: 6OIJ) as a template ^24–26^ (Fig. 2A). These predicted structures were embedded in a phosphatidylcholine lipid bilayer using the Bilayer Builder tool in CHARMM-GUI^27^.

### Molecular dynamics simulation and clustering

Each complex was relaxed and equilibrated through 500-ns NPT ensemble conventional molecular dynamics (cMD) simulations using a 2-fs time step, an AMBER force field, and AMBER’s pmemd.cuda program^44^. For the duration of all simulations, pressure and temperature were kept at 1 atm and 310.15 K, respectively. Before full-length cMD simulations, both complexes underwent six relaxation steps to minimize energy and equilibrate the systems as described previously^28^. G_12_D–Gα_12_ and G_12_D–Gα_13_ complexes were then subjected to 50-ns GaMD equilibration followed by a 500-ns GaMD production run using default parameters, which includes harmonic dual-boost potential to reduce energy barriers to enhance conformational sampling^29^. To find distinct conformations in the GaMD snapshots, the DBScan clustering algorithm was used with the “*minpoints*” parameter set to 4 conformations and the distance cutoff “*epsilon*” parameter of 2.25 Å^45^. The full receptor–Gα complex was aligned during clustering. The DBScan clustering algorithm used pairwise-RMSD of this complex between any two trajectory snapshots as the distance metric for clustering. Any two conformation with an RMSD greater than 2.25 Å is put in different clusters and all final reported clusters should have at least 4 conformations in each cluster based on the parameters used. The advantage of this algorithm over other popular algorithms like K-means clustering and hierarchical clustering is that in DBScan clustering one does not need to specify the target number of clusters, because the number of clusters should be determined by the underlying dynamics of the molecular system. The conformation that best represented each DBScan cluster as its centroid was chosen as a starting point for an additional 500-ns cMD simulation to allow for unbiased MMPBSA energy calculations and analysis of receptor–G-protein interactions. Residue interaction analysis was performed on the average structure of the most populated cluster for each complex.

### Receptor–Gα binding free energy analysis

MMPBSA-based total energy (∆G) was determined by calculating the difference between the complex free energy and the sum of the G protein and receptor free energies as described previously^28^.

### Data analysis

Concentration–response curves were fitted using Nonlinear Regression Analysis: Variable slope (four parameters) function in the GraphPad Prism 9 software (GraphPad) was used with absolute Hill Slope values less than 2. Sigmoid maximum effect (*E*_*max*_, also referred to as Span) and the negative log of the half-maximal excitation concentration (pEC_50_) were used to evaluate G-protein-coupling activity. Protocols for normalizing and for generating experimental replicates are described in the legends of each Figure, as appropriate.

### Supplementary Information

Supplementary Information.

Supplementary Figures.

## Data Availability

All data generated or analyzed in study are provided in the separated Source Data file in Supplementary information file section.
