# Supplementary material for: Identification of Gα12-vs-Gα13-coupling determinants and development of a Gα12/13-coupled designer GPCR
Source: Sci Rep. 2024 May 15;14:11119. doi: 10.1038/s41598-024-61506-4 (PMC11096383; doi:10.1038/s41598-024-61506-4)
Supplement: Supplementary file 2 — Supplementary Figures. [file 41598_2024_61506_MOESM2_ESM.pdf]

Supplementary Figure 1

Expression levels of $G\alpha_{q\text{-}12C}$ and $G\alpha_{q\text{-}13C}$ mutants

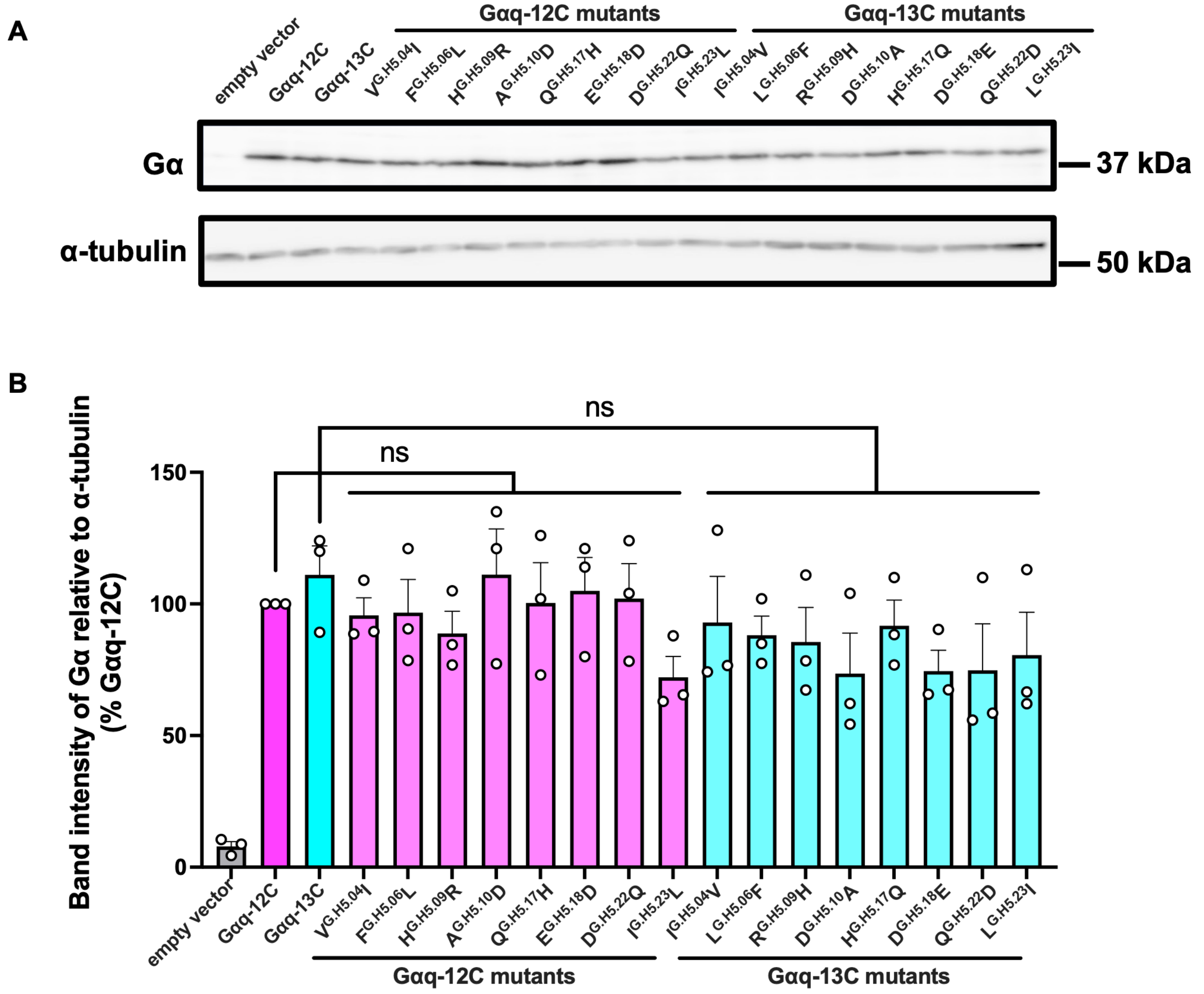

(A) Western blot analysis of $G\alpha_{q\text{-}12C}$, $G\alpha_{q\text{-}13C}$ and their mutants expressing in $\Delta G_q/\Delta G_{12}$ cells along with G_{12}D and AP-TGFα. α-tubulin was used as a loading control. The original blots are presented in the Supplementary source data file.

(B) Densitometry quantification of the western blot analysis. For individual samples, intensities of Gα band intensities were normalized by those for α-tubulin. Bars and error bars represent the mean and SEM, respectively, for three independent experiments with each dot representing an individual experiment. ns represents $P > 0.05$ with one-way ANOVA, followed by Tukey's multiple comparisons test.

Supplementary Figure 2

Coupling activity of $G\alpha_{q\text{-}12C}$ and $G\alpha_{q\text{-}13C}$ mutants to $G_{12}D$

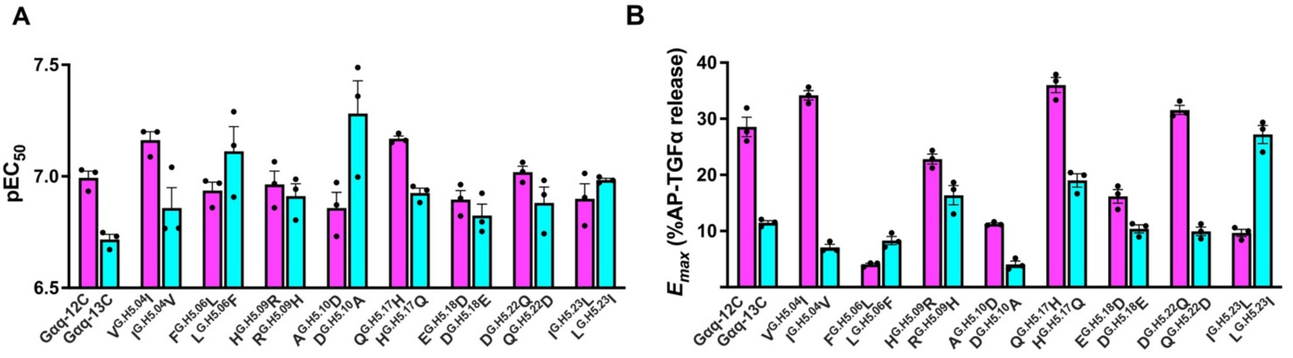

(A, B) pEC_{50} and E_{max} values obtained from Figure 1B and F. Bars and error bars represent the mean and SEM, respectively, for three independent experiments with each dot representing an individual experiment.

Supplementary Figure 3

Trajectory and distribution analyses of the cMD simulations

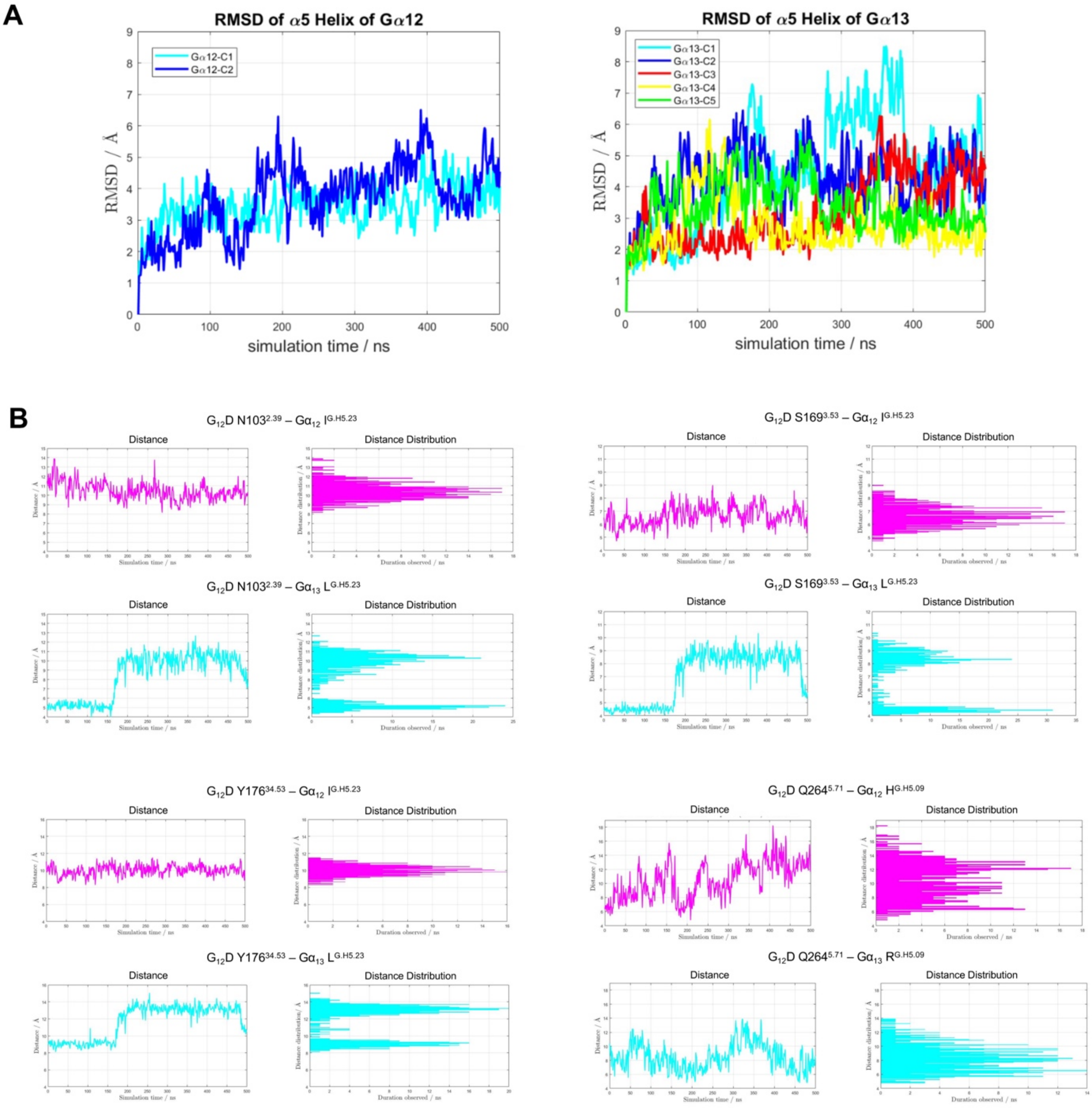

(A) RMSD plots for α5 helix in cMD simulations for all clusters. The reference poses corresponded to the first frame in each of the simulations to capture the dynamics within each cluster.

(B) Distance plot trajectories and distribution histograms of the indicated residues during the 500-ns cMD simulations corresponding to the C1 cluster from GaMD simulations. The initial poses of the $G_{12}D$-$G\alpha_{12}$ and the $G_{12}D$-$G\alpha_{13}$ complexes were obtained from the most populated clusters (C1) observed in the GaMD simulations.

Supplementary Figure 4

Expression levels of $G_{12}D$ mutants

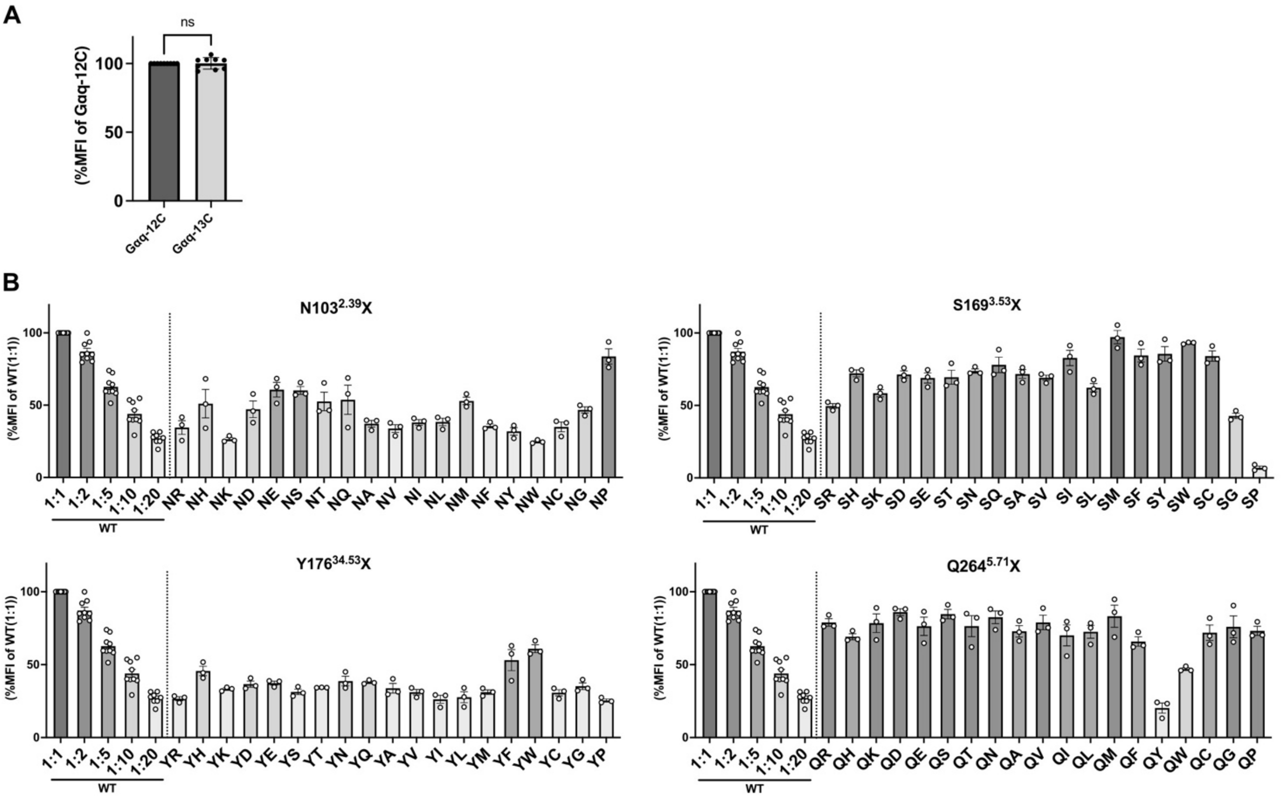

(A) Cell-surface expression levels of $G_{12}D$. $\Delta G_q/\Delta G_{12}$ cells transiently expressing N-terminally FLAG-tagged $G_{12}D$ and with $G\alpha_{q\text{-}12C}$ or $G\alpha_{q\text{-}13C}$ and were subjected to the flow cytometry analysis. There is no significant difference in the expression level of $G_{12}D$ between the condition with $G\alpha_{q\text{-}12C}$ and the condition with $G\alpha_{q\text{-}13C}$. ns represents $P > 0.05$ with the two-tailed t-test. MFI, mean fluorescent intensity.

(B) Cell-surface expression levels of the $G_{12}D$ mutants transiently expressed in $\Delta G_q/\Delta G_{12}$ cells along with $G\alpha_{q\text{-}12C}$. Expression levels of the mutants were normalized to that of WT. The data for WT and its dilutions are reused in all panels.

In all panels, bars and error bars represent the mean and SEM, respectively, for 3-9 independent experiments with each dot representing an individual experiment.

Supplementary Figure 5

$G\alpha_{q\text{-}12C}$- and $G\alpha_{q\text{-}13C}$-coupling activity of $N103^{2.39}$ mutants

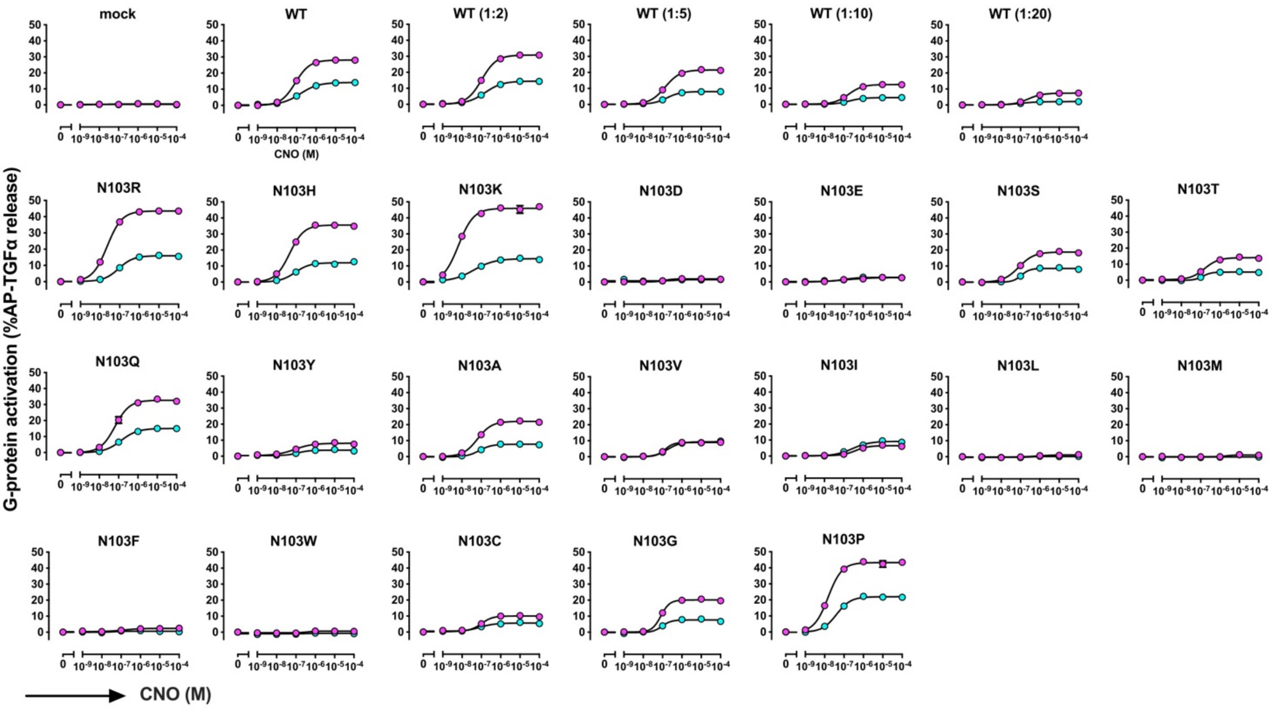

Concentration–response curve for the TGFα shedding responses of the representative WT and $N103^{2.39}$ mutants. In all panels, the symbols and error bars represent the mean and SEM, respectively, for 3-15 independent experiments. For many data points, the error bars are smaller than the symbols and, thus, are not visible.

Supplementary Figure 6

G$\alpha_{q\text{-}12C}$- and G$\alpha_{q\text{-}13C}$-coupling activity of S169$^{3.53}$ mutants

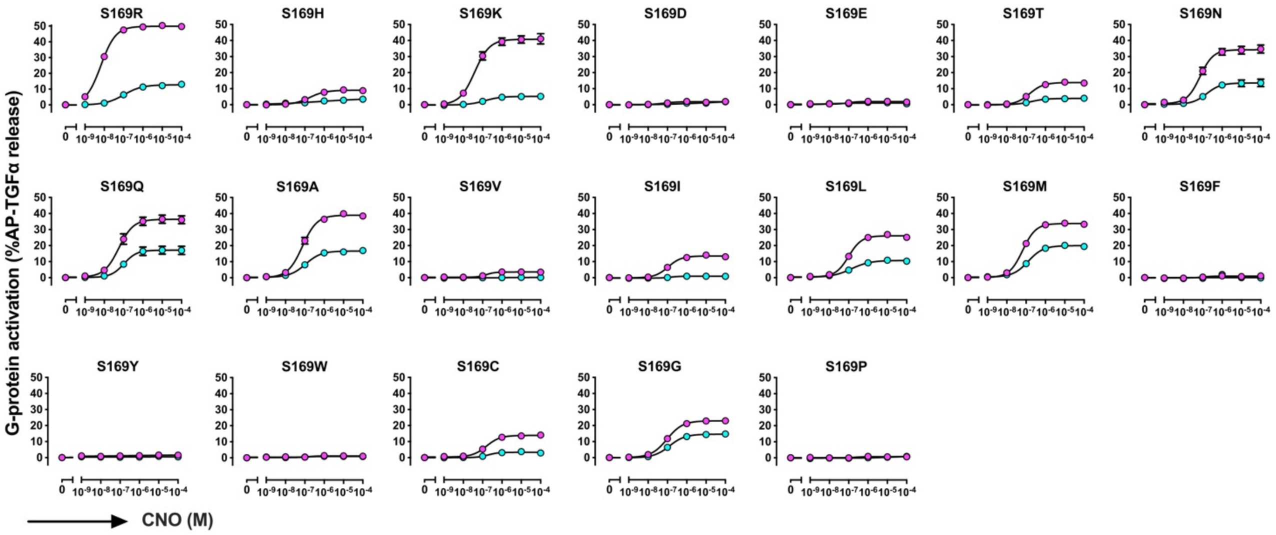

Concentration–response curve for the TGFα shedding responses of the representative S169$^{3.52}$ mutants. In all panels, the symbols and error bars represent the mean and SEM, respectively, for three independent experiments. For many data points, the error bars are smaller than the symbols and, thus, are not visible.

Supplementary Figure 7

G$\alpha_{q\text{-}12C}$- and G$\alpha_{q\text{-}13C}$-coupling activity of Y176$^{34.53}$ mutants

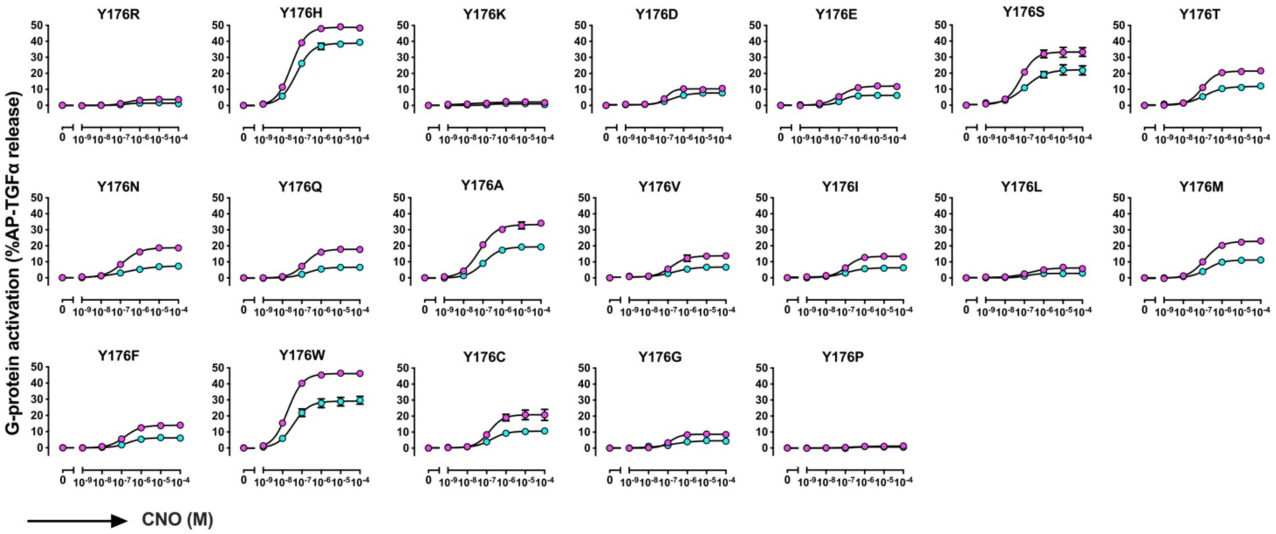

Concentration–response curve for the TGFα shedding responses of the representative Y176$^{34.53}$ mutants. In all panels, the symbols and error bars represent the mean and SEM, respectively, for 3-6 independent experiments. For many data points, the error bars are smaller than the symbols and, thus, are not visible.

Supplementary Figure 8

G$\alpha_{q\text{-}12C}$- and G$\alpha_{q\text{-}13C}$-coupling activity of Q264$^{5.71}$ mutants

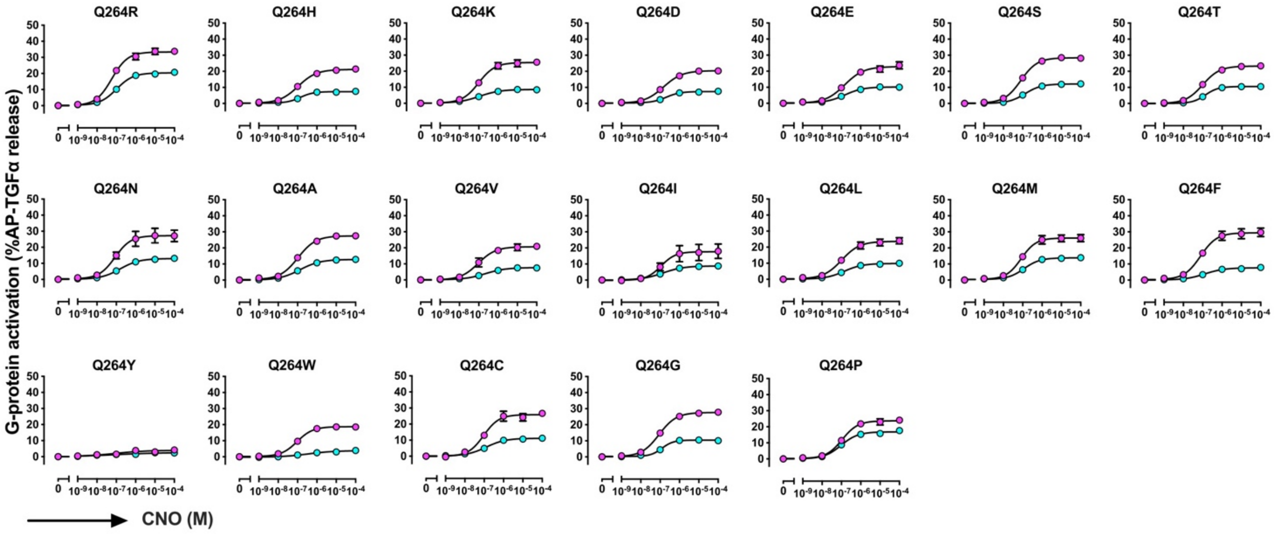

Concentration–response curve for the TGFα shedding responses of the representative Q264$^{5.71}$ mutants. In all panels, the symbols and error bars represent the mean and SEM, respectively, for three independent experiments. For many data points, the error bars are smaller than the symbols and, thus, are not visible.

Supplementary Figure 9

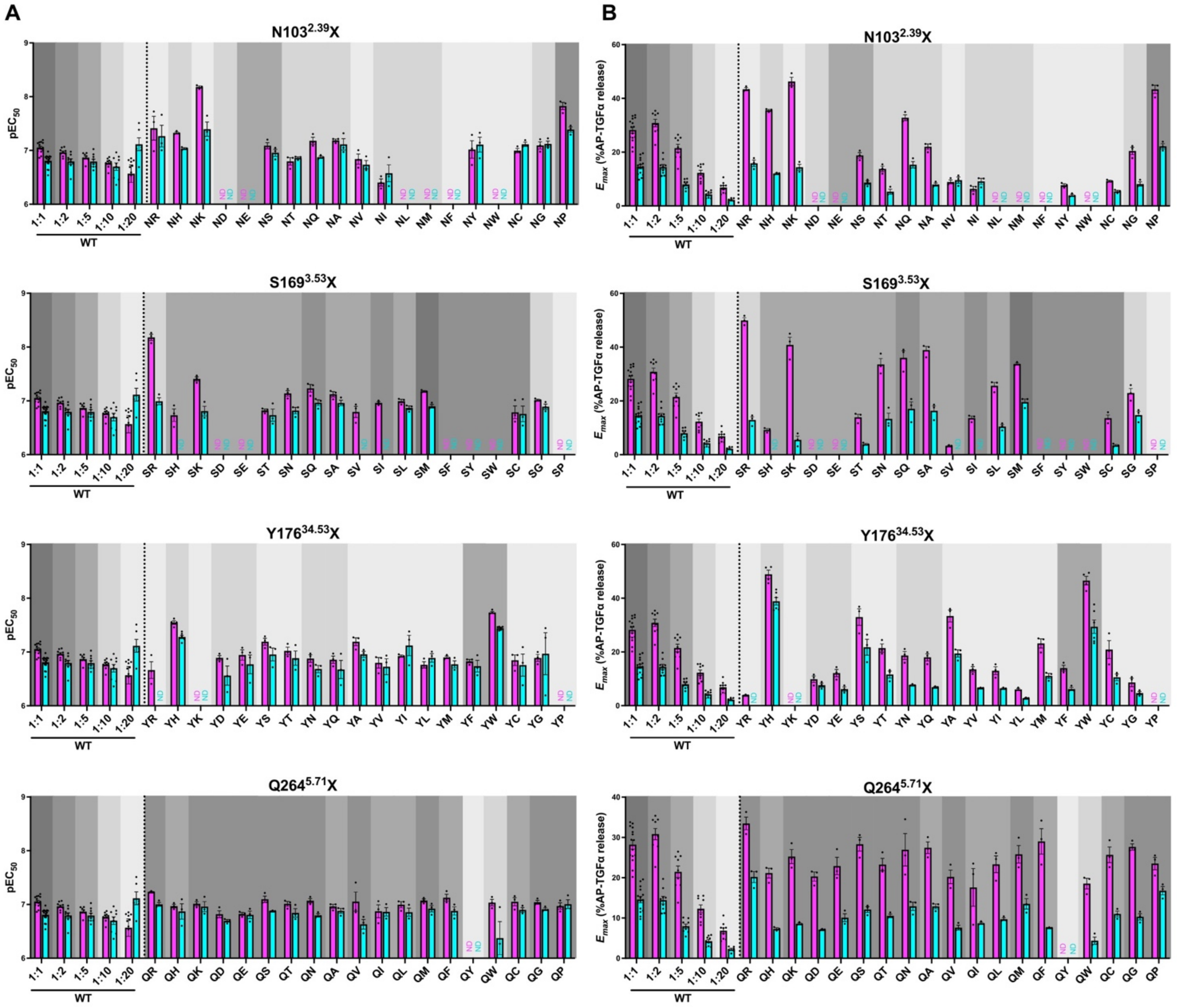

G$\alpha_{q\text{-}12C}$- and G$\alpha_{q\text{-}13C}$-coupling activity of the $G_{12}D$ mutants

(A, B) pEC_{50} and E_{max} values obtained from Supplementary Figure 5-8. Bars and error bars represent the mean and SEM, respectively, for 3-15 independent experiments with each dot representing an individual experiment. The data for WT and its dilutions are reused in all panels.

Supplementary Figure 10

Expression levels and coupling activity of the dual mutants

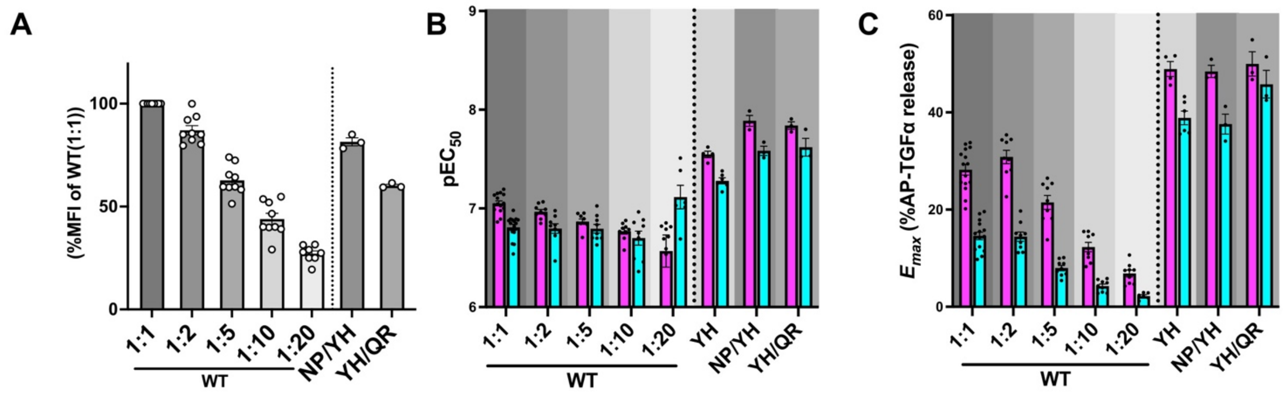

(A) Cell-surface expression levels of N103$^{2.39}$P/Y176$^{34.53}$H and Y176$^{34.53}$H/Q264$^{5.71}$R. $\Delta G_q/\Delta G_{12}$ cells transiently expressing N-terminally FLAG-tagged $G_{12}D$ and with $G\alpha_{q\text{-}12C}$ or $G\alpha_{q\text{-}13C}$ and were subjected to the flow cytometry analysis. Bars and error bars represent the mean and SEM, respectively, for 3-9 independent experiments with each dot representing an individual experiment. MFI, mean fluorescent intensity. The data for WT and its dilutions are reused from Supplementary Figure 4.

(B) pEC_{50} and E_{max} values obtained from Figure 5B. Bars and error bars represent the mean and SEM, respectively, for three independent experiments with each dot representing an individual experiment. The data for WT and its dilutions are reused from Supplementary Figure 9.
